# Physics of Brain Cancer: Multiscale Alterations of Glioblastoma Cells under Extracellular Matrix Stiffening

**DOI:** 10.3390/pharmaceutics14051031

**Published:** 2022-05-10

**Authors:** Mohammad Khoonkari, Dong Liang, Marleen Kamperman, Frank A. E. Kruyt, Patrick van Rijn

**Affiliations:** 1 Department of Medical Oncology, University of Groningen, University Medical Center Groningen, Hanzeplein 1, 9713 GZ Groningen, The Netherlands; m.khoonkari@rug.nl (M.K.); d.liang01@umcg.nl (D.L.); 2 Polymer Science, Zernike Institute for Advanced Materials, University of Groningen, Nijenborgh 4, 9747 AG Groningen, The Netherlands; marleen.kamperman@rug.nl; 3 Department of Biomedical Engineering-FB40, University of Groningen, University Medical Center Groningen, A. Deusinglaan 1, 9713 AV Groningen, The Netherlands; 4 W.J. Kolff Institute for Biomedical Engineering and Materials Science-FB41, University of Groningen, University Medical Center Groningen, A. Deusinglaan 1, 9713 AV Groningen, The Netherlands

**Keywords:** physics of cancer, glioblastoma multiforme, extracellular matrix stiffening, tumor microenvironment, mechanical stress, adaptive cellular signaling

## Abstract

The biology and physics underlying glioblastoma is not yet completely understood, resulting in the limited efficacy of current clinical therapy. Recent studies have indicated the importance of mechanical stress on the development and malignancy of cancer. Various types of mechanical stress activate adaptive tumor cell responses that include alterations in the extracellular matrix (ECM) which have an impact on tumor malignancy. In this review, we describe and discuss the current knowledge of the effects of ECM alterations and mechanical stress on GBM aggressiveness. Gradual changes in the brain ECM have been connected to the biological and physical alterations of GBM cells. For example, increased expression of several ECM components such as glycosaminoglycans (GAGs), hyaluronic acid (HA), proteoglycans and fibrous proteins result in stiffening of the brain ECM, which alters inter- and intracellular signaling activity. Several mechanosensing signaling pathways have been identified that orchestrate adaptive responses, such as Hippo/YAP, CD44, and actin skeleton signaling, which remodel the cytoskeleton and affect cellular properties such as cell–cell/ECM interactions, growth, and migration/invasion of GBM cells. In vitro, hydrogels are used as a model to mimic the stiffening of the brain ECM and reconstruct its mechanics, which we also discuss. Overall, we provide an overview of the tumor microenvironmental landscape of GBM with a focus on ECM stiffening and its associated adaptive cellular signaling pathways and their possible therapeutic exploitation.

## 1. Introduction: Glioblastoma

Glioblastoma multiforme (GBM) is the most aggressive and malignant type of brain tumor [1]. A combination of surgery, chemotherapy, and radiotherapy result in a median survival of around 16 months due to the failure to remove the whole tumor and therapy resistance leading to a deadly tumor relapse [2]. Moreover, there are still many blind spots regarding the characteristics and properties of GBM cells and their tumor microenvironment (TME), which drives tumor progression. There is a great need to develop better treatments for GBM. However, many new drugs and treatment strategies that have activity in other tumor types fail in GBM, and therefore a better fundamental understanding of the biology driving GBM is needed.

GBM occurs most often in the cerebral hemispheres, especially in the frontal and temporal lobes of the brain, where it expands very fast. Being a semi-solid and highly heterogeneous tumor, GBM has several unique physiochemical, mechanical, and biological features [3,4]. Recent studies showed that the extracellular matrix (ECM) of the brain and its alterations around the GBM tumor niche are directly linked to the rapid progression of GBM [4,5]. In fact, upon genetic mutation–driven GBM tumor formation, changes simultaneously occur in the TME, including in the ECM [6]. These local alterations within the TME later expand to the tissues of the full [7,8]. This series of gradual changes biologically alter GBM cells and their properties involving reciprocal ECM–GBM cell interactions [8]. Whether a cancer promoting TME is the outcome of abnormal cell mutation or the other way around is debatable, but it is clear that they coevolve during tumorigenesis [8,9]. For the ECM, in the initial stages of GBM, local ECM alterations occur that expand across the brain, and in later stages these alterations also facilitate tumor invasion [10,11]. The ECM alterations in GBM mostly comprise altered expression levels of several components. These result in increased tissue stiffness that can affect rapid biological and physiological changes in the cellular activities that stimulate tumor development and progression [12,13,14]. A stiffening of the ECM is one of the mechanical components that impact cancer progression in the field of cancer physics [15,16,17].

## 2. Physics of Cancer

**Figure 1:**
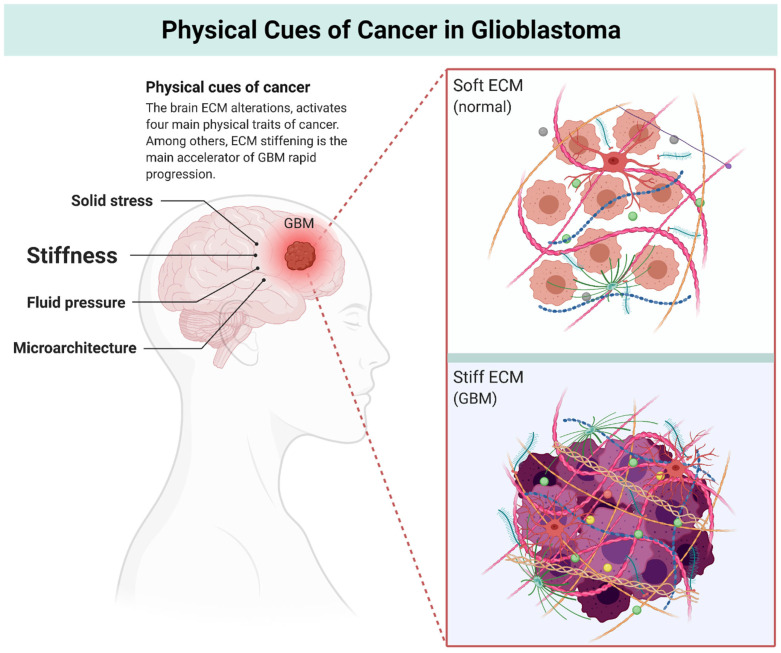
Physical traits of cancer. Solid stress, stiffness, fluid pressure, and microarchitecture are the four distinct physical cues which extensively drive GBM tumor progression. Among others, Extracellular matrix (ECM) stiffening directly links with the glioblastoma (GBM) stem cells invasiveness and motility. Figure is adapted from [15,20,22,23].

Cancer is initiated by genetic modifications, but develops by altering its physical context [18]. Through a process known as mechanotransduction, cells sense their microenvironment and adapt to it by modifying their cellular and extracellular structures [19]. There is increasing recognition that mechanical forces play a crucial role in many of the hallmarks of cancer [15]. However, the cellular and extracellular changes by which tumor cells adapt to mechanical forces are often overlooked and incompletely understood, as is the potential involvement of specific oncogenes. Recently, the physical traits of cancer, which consist of solid stress, stiffness, fluid pressure, and microarchitecture were reviewed (Figure 1) [20]. The multidisciplinary field of the physics of cancer studies how mechanical and chemical cues orchestrate the adaptive response of (cancer) cells to ECM alterations and, generally, tissue alterations [21]. Better knowledge of such mechanisms may open new doors to develop novel therapeutics for cancer.

## 3. Multiscale Alterations of the Brain ECM during GBM Progression: Turning Soft into Stiff

Cells are embedded within an ECM, a gel-like substance that serves as a matrix to which cells adhere to and support cell viability and proliferation [24]. The ECM of the brain is primarily made up of (i) polysaccharides such as glycosaminoglycans (GAGs); (ii) proteoglycans; (iii) fibrous proteins (glycoproteins) such as collagen, elastin, fibronectin, and laminin; and (iv) many types of growth factors [25].

(i) GAGs are sugar molecules linked together by amino acids, which makes them repeating disaccharide units in which one is an amino sugar. Examples of GAGs include keratin sulfate, heparin sulfate, dermatin sulfate, and the most important one, hyaluronan or hyaluronic acid (HA) (non-sulfated). These sugars carry negative charges through a sulfate (SO_3_^−^) or carboxyl group (COO^−^). These negative charges make GAGs the most anionic molecules in mammalian cells [26]. GAGs are capable of high water retention due to their negative charges. The function of GAGs is mainly to regulate activity of secreted proteins and to immobilize secreted molecules close to where they are secreted to provide a reservoir of proteins for later use. They also play a role in tissue repair processes, including angiogenesis. Moreover, GAGs protect proteins from proteolytic degradation and alter or concentrate proteins for presentation to cell surface receptors [12,25,26].
(ii) Proteoglycans are proteins that are heavily glycosylated. The basic proteoglycan unit consists of a core protein with one or more covalently attached GAG chains [27]. The chains are long, linear carbohydrate polymers that are negatively charged under physiological conditions due to the presence of sulfate and uronic acid groups. Proteoglycans are a major component of the ECM, i.e., the filler substance between the cells in an organism [28]. They form large complexes, both to other proteoglycans, such as hyaluronan, and to fibrous matrix proteins, such as collagen. They are also involved in binding cations such as sodium, potassium, and calcium, as well as water, but also regulate the movement of molecules through the matrix [25,28]. Evidence also shows they can affect the activity and stability of proteins and signaling molecules within the matrix [29]. The individual functions of proteoglycans can be attributed to either the protein core or the attached GAG chain. The most common type of proteoglycans within the brain ECM are aggrecan, brevican, glypican-1, versican, and tenascin-C [8,25].
(iii) Glycoproteins provide structure and adhesive functions for the cells [12,25,30]. They have direct or indirect links with most of the intercellular and intracellular signaling pathways [10]. Collagen is one of the most important fibrous proteins, consisting of covalently intermolecular and intramolecular cross-linked helices. These helices are composed of hydroxyproline and hydroxylysine [31]. It is reported that collagen directly affects the ECM structure [8]. While fibrillar collagens are the most abundant proteins in the body and are highly expressed in the interstitial matrix of several organs, the normal adult brain contains very limited amounts of collagen, accounting for its soft consistency. The major form of collagen in the brain is collagen IV, which is present in the basement membrane surrounding the vascular endothelial cells. Collagen IV levels are upregulated in gliomas and localized to the basement membrane lining the vessel walls in astrocytomas of all grades, including GBM. Whether fibrillar collagens are present in gliomas is more contentious, and it has been reported that GBMs do not express intratumoral fibrillar collagen. The identity of the collagen producing cells is still unresolved but glioma cells can synthesize their own complement of ECM, including collagens I and IV [32]. During GBM progression, the collagen content of the brain does not increase rapidly and is reported to be tenfold lower compared to the GAGs. Therefore, collagen does not contribute to the ECM stiffening and generation of mechanical stress in GBM. However, aligned collagen fibers increase the presence of confined spaces within the brain and could fuel the migratory tendency of glioblastoma stem cells (GSCs) [33]. Elastin, another fibrous protein within the brain ECM, is a highly hydrophobic elastic protein secreted and organized in fibers and sheets and provides protection against tensile forces [34]. Fibronectin helps cells to attach to the matrix by first attaching to cells through its multiple binding domains via the RGD sequence [35,36].
(iv) A wide variety of growth factors present within the ECM also continuously affect cell behavior by promoting cell adhesion, growth, proliferation, and differentiation during nervous system development. For instance, epidermal growth factor (EGF) is a polypeptide that acts as a signaling molecule in initiating mitosis and promotes rapid cell growth. Cytokine vascular endothelial growth factor (VEGF) controls brain angiogenesis and vascular network formation within the brain ECM. Fibroblast growth factors (FGF) regulate embryonic development, organogenesis, and tissue differentiation. Brain-derived growth factor (BDNF) serve as a survival factor during early ECM development [37].

**Figure 2:**
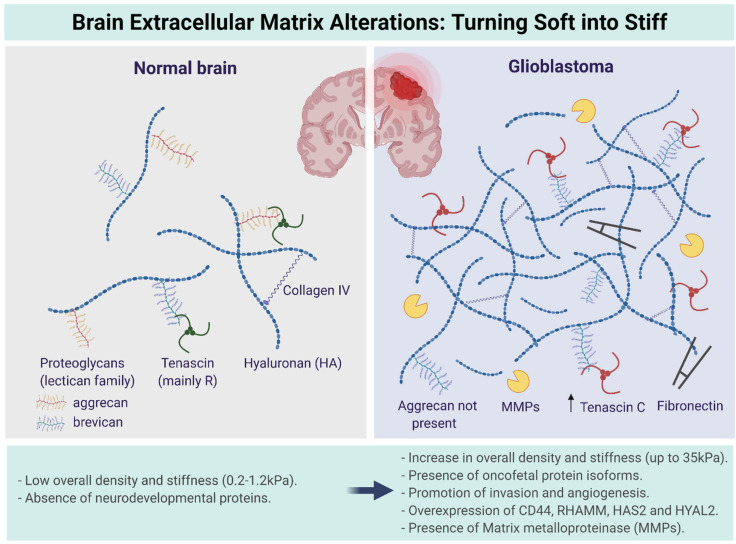
Overexpression of the brain extracellular matrix (ECM) components drastically alters its composition, mechanics, and physiochemical properties. Increased expression of hyaluronic acid, tenascin-C, fibronectin, and brevican, stiffens the ECM, which generates mechanical stress. Increased expression of HA-related genes such as CD44, RHAMM, and HAS2, intensifies HA overexpression. In addition, elevated presence of MMPs initiates matrix protein degradation, which weakens the ECM opposing the stiffening phenomena. Figure adapted from [12,25,38,39].

Several of the listed brain ECM components undergo overexpression during GBM tumor progression, which is known to be the main cause of ECM stiffening. Figure 2 highlights the most important ECM components involved in ECM stiffening and demonstrates the ECM compositional differences between a healthy ECM and the GBM brain’s ECM. Particularly, the overexpression of HA, tenascin-C, fibronectin, and brevican within the GBM ECM, coupled with increased expression of HA-related genes such as CD44 and RHAMM, drive the stiffening phenomena.

Many functions of the matrix also involve cell adhesion molecules (CAMs). CAMs are integral cell membrane proteins that mediate cell–cell and cell–matrix adhesion [40]. Among all types of CAMs, integrins and cadherins are the most important ones that bind directly to the ECM and cell cytoskeleton components, whereas integrins are the most important for mediating cell–matrix adhesion [41]. Thus, many ECM components have an effect on cell–cell and cell–ECM interactions, and as mentioned earlier, show GBM-localized alterations in expression that can extend to surrounding tissue [38]. GBM massively changes the ECM harmony within normal brain tissue, resulting in an abnormal ECM environment that favors GBM progression and invasion, which is mediated by activation of specific signaling pathways [42]. Therefore, cell–ECM interactions are closely monitored by cells that in turn activate adaptive responses to balance such ECM alterations.

HA, glypican-1, brevican, neurocan, tenascin-C, and versican are all overexpressed within the ECM of GBM with gradual tumor growth [38]. In fact, high HA expression is one of the most important ECM alterations, with multifactorial functions, and is discussed further below [43]. HA overexpression significantly alters the mechanics of the brain tissue [44]. The increase in collagen expression promotes aligned microarchitecture within the brain ECM structure [45]. Fibronectin expression also increases and promotes cell adhesion properties [46]. Moreover, matrix metalloproteinase (MMPs) activity is facilitated by an altered ECM of GBM, initiating ECM protein degradation, which weakens the ECM’s mechanical properties that oppose the stiffening phenomena [47]. On the contrary, the aggrecan concentration within the GBM ECM is decreased [29,38]. These massive compositional alterations directly affect the physicochemical properties of the brain ECM and initiate gradual ECM stiffening. Normal brain ECM has a stiffness of 0.2 to 1.2 kPa, which increases up to 45 kPa during GBM tumor development [48,49]. Such a sharp increase in matrix stiffness activates the mechanotransduction process in GBM cells [20,50,51].

### The Multifunctional Role of Hyaluronic Acid in ECM Alterations

HA is a repeating disaccharide unit of N-acetylglucosamine and glucuronic acid [52]. The central nervous system (CNS), which includes the brain and spinal cord, contains a high HA level [53]. The brain ECM is composed of 25% HA in mass, which gradually increases during GBM tumor progression [53,54,55], making HA-enriched ECM the most common feature of the GBM TME [38,56]. 

HA interacts with proteins and other GAGs via unique binding sites and various linker proteins, forming a complex mesh [57]. HA directly participates in diverse biological processes, including inflammation, angiogenesis, and tissue regeneration. HA affects the proliferation and motility of GSCs as well as that of neural stem cells (NSCs) [57]. HA asserts its biological functions via several non-integrin cell surface receptors, which include CD44 (HA interaction via amino-terminal HA-binding region of CD44), the receptor for hyaluronan-mediated motility (RHAMM), lymphatic vessel endothelial hyaluronan receptor 1 (LYVE-1), intracellular adhesion molecule 1 (ICAM-1), and Toll-like receptors (TLRs) 2 and 4 [56]. In the CNS, increased astrocytic expression of CD44 appears to be an essential response to injury [57,58]. HA levels in the ECM are regulated by a balance of HA degradation by hyaluronidases, receptor-mediated endocytosis of extracellular HA (HYAL-1, HYAL-2 and HYAL-3), and direct deposition of new HA into the ECM by HA synthases (HAS-1 and HAS-2) [56]. 

**Figure 3:**
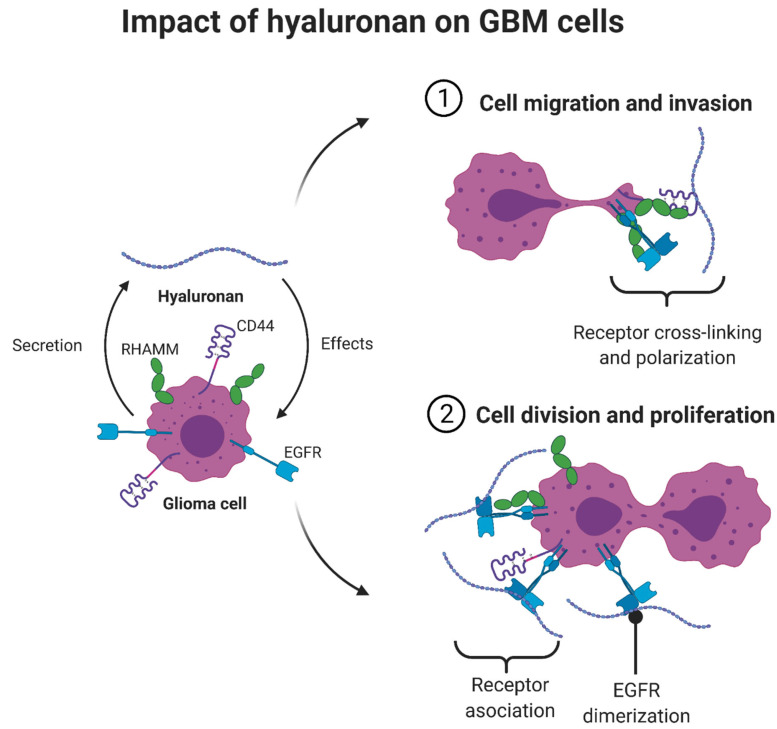
Impact of hyaluronic acid (HA) on GBM cells. HA, through increased expression of CD44 and RHAMM, coupled with active EGFR (dimerized), facilitates GBM cell motility, division, and proliferation. Figure adapted from [44,57,59,60,61].

The HA content of the ECM is coupled to cellular morphological changes and F-Actin expression, which results in facilitating cell movements. Increased HA expression leads to ECM stiffening, which applies mechanical stress on GSCs and stimulates F-actin expression. Additionally, increased expression of CD44, improves cell adhesion, which facilitates GSC motility [12,57]. Figure 3 summarizes the multifunctional effect of HA on GBM cells.

To better understand the role of HA in GBM, in vitro models have been developed using hydrogels to investigate the adaptive GSC responses. Cha et al. [43,56] used HA–collagen-based hydrogels with varying HA contents to investigate the effect of HA-enriched matrices on GBM cells. Using spheroids formed with GBM cells, they observed higher cell proliferation by increasing the HA level within the gel matrix. In addition, they showed an enhanced migratory state of GBM cells within gels with a higher HA content. This showed that overexpression of HA within the brain ECM facilitates GBM cell motility and proliferation [62]. Chen et al. [44] investigated the effect of HA’s molecular weight (Mw) on the activation of HA-related receptors on cells in GBM and its role in GBM cell malignancy. Since hyaluronidase enzymes (HYAL-1 and HYAL-2) secretion from GBM cells increases within the altered ECM, upon HA degradation, short and long-chain HA are both present within the ECM, which results in a variation in HA’s Mw. This change regulated the HA-related cell receptors, especially CD44. It has been reported that GBM cells cultured in hydrogels containing 500 kDa matrix-immobilized HA, with controlled physical properties, showed less invasive potential than those in hydrogels containing matrix-immobilized 10 or 60 kDa HA. This increased malignancy seems to be related to different interrelated factors: cell-secreted HA, matrix degradation, and cell–matrix signaling. Erickson et al. [63] used an HA-based hydrogel to culture GBM cells in 3D. By developing a complex polyelectrolyte scaffold based on HA and different concentrations of chitosan, they were able to mimic the matrix stiffening in GBM and showed how a high level of matrix stiffness advances GBM cell proliferation. They also showed that the increase in matrix stiffness, induced by HA, stimulates the expression of MMPs, hyaluronidases, and CD44 from GBM cells. It shows that, along with HA overexpression and its influence on CD44, matrix rigidity intensifies such signaling as well. Lou et al. [64] showed that an HA-enriched matrix promotes cell spreading and activates F-actin polymerization. These results highlight that HA is involved in cell elongation and morphological alteration. Zamboni et al. reported that HA increases cell viability and, upon its overexpression, develops matrix adhesion properties through stimulating CD44 expression and integrin-mediated adhesion [65]. 

## 4. Mechanics of the Brain and GBM Cells

The brain is surrounded by the skull as a solid barrier, and, upon GBM development, the size of the brain increases gradually as the tumor grows, leading to increasing intracranial pressure (ICP) [66]. ICP, which is around 17–19 mmHg at rest, rises to 25 mmHg in the later stages of GBM, initiating direct mechanical stresses and solid forces from the brain tissue to the skull and vice versa [20,67]. It is reported that the water content of the brain during GBM progression slightly increases, [68,69] which is known as one of the associated outcomes of the ECM alterations [70,71] and leads to cerebral edema (brain swelling) [69,72,73]. Recent studies via magnetic resonance (MR) elastography of the brain during GBM progression confirm the mentioned trends [74,75]. Cerebral edema, together with ECM stiffening, can also raise the inner pressure of the brain, leading to rearrangement of the ECM microarchitecture [20]. GBM is constituted not only by highly proliferative malignant astrocytoma cells but also by immune cells, both residing in and infiltrating stromal cells, vascular endothelial cells, and pericytes, which all create separate niches within the tumor. All these cells are able to interact with each other within the ECM. Although intratumor heterogeneity as a concept is often restricted to the varying presence of the different genetic alterations present in the different tumor cells, the true heterogeneity probably far exceeds this level as many intratumoral niches can be defined based on the relative composition of contributing cell (sub-) types and ECM substances. In these niches, different tumor cell types (proliferating, infiltrating, CSC like) and different noncancerous cells (microglia, macrophages, dendritic cells (DCs), lymphocytes) dynamically reshape different parts of the tumor, and it is not clear which are the key cell types in malignant progression and ECM alteration [76]. Microscopically, this results in different microenvironments within the tumor, varying from solid tumor cores with densely packed proliferating tumor cells, to necrotic and perinecrotic areas, perivascular areas around vessels with endothelial proliferation, and hypoxic and perihypoxic regions, while all of these regions are ruled by the microclimates of cells and molecules [76]. While all of these cell types hold specific functions, it is mainly CSCs that orchestrate the ECM stiffening phenomena through direct intracellular interactions with the ECM’s overexpressed components, such as GAGs and proteoglycans [76]. Therefore, in the context of the physics of cancer, GSCs are of prime interest to be studied; while studying other cell types could contribute to the understanding drug resistance in GBM, such factors are outside the scope of this review. 

### The Brain ECM under Tension: A Look into the Brain ECM Microarchitecture

**Figure 4:**
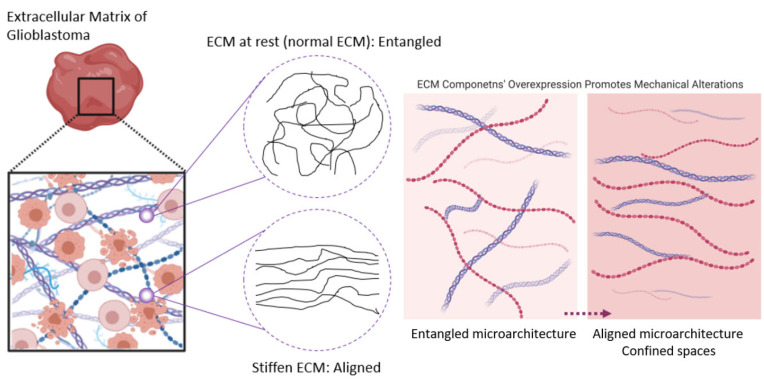
The brain ECM mechanics and microarchitecture in GBM. With extracellular matrix (ECM) alterations, as its stiffens, the brain tissue structure adopts confined spaces and reshapes its microarchitecture, generating compression and applying mechanical stresses to GSCs. Figure adapted from [73,78,80,81,82,83].

The stiffening of the brain ECM exerts a high load of mechanical stress, which alters GBM cell behavior through a series of mechanoresponsive, adaptive cellular signaling pathways [77]. In fact, GBM cells sense the matrix stiffening as a type of applied mechanical stress, which is why ECM stiffening phenomena is most often read as mechanical stress [78]. In GBM, ECM alterations lead to its stiffening. As the ECM stiffens, along with an increased expression of its fibrous proteins, its structure reforms by adopting an aligned microarchitecture and creating confined spaces (Figure 4) [78,79,80]. Confined spaces enhance the migratory state of the GBM cells, where increased stiffness directly mediates the motility and invasion of GBM cells [81].

Mechanical stress is initiated by matrix stiffening, and, upon its initiation, it can propagate throughout the brain ECM and fuel the stiffening (its source), while solid stress is initiated by tumor growth (size increase), i.e., reflection forces from the skull to the tissue and brain swelling (cerebral edema) [18,20]. With respect to the physical traits of cancer, both types of mentioned stresses are present within the ECM of GBM. Nia et al. [84], with a novel approach, showed the effect of solid stress (localized applied force) on a mouse brain with an engineered in vivo compression setup. The setup directly applied solid stress to the cortex and cerebellum parts of the mouse brain, and the resulting alterations were investigated via advanced imaging techniques. This setup was used to mimic the tumor growth over time and study its impacts on the brain ECM. They reported how chronic compression results in cellular responses, quantified via histological and molecular techniques. They reported that in response to compression, there are fewer perfused vessels, astrocytes become activated (GFAP), loss of neuronal network formation occurs, gene expression of TNF-α increases, and there is chromatin condensation and the activation of calcium ion channels [85,86]. 

## 5. Mechanobiology of GBM Cells: Adaptive Cellular Signaling Pathways

As the ECM stiffens, the associated mechanical changes are recognized by mechanosensors in the cell that transmit forces via chemical signals [87,88]. To mend the balance between internal and external rigidities, the cell activates adaptive/responsive signaling pathways that increase contractility and henceforth reinforce the cytoskeleton [87].

As the ECM stiffens, a sharp increase in focal adhesion assembly comes first, followed by advanced cell proliferation [89,90]. Additionally, the increasing basement membrane stiffness leads to malignant transformation. Applying mechanical stress to integrins induces Rho signaling, which activates g-actin polymerization, leading to F-actin filament assembly [91]. 

Yes-associated protein (YAP) and transcriptional coactivator with PDZ-binding motif (TAZ) localize to the nucleus in GSCs cultured on a stiff substrate, while they remain in the cytoplasm when cells are cultured on a soft matrix. YAP and TAZ promote proliferation as it localizes within the nucleus, and, at elevated levels, it can result in neoplasia [92]. ECM stiffening with the generation of mechanical stresses in the ECM propagates along cytoskeletal filaments and reaches the nucleus, affecting gene expression and the integrity of the genome. Nuclear distortion, including spindle and chromosome rearrangement, occurs due to the reorganization of cytoskeletal filaments induced by mechanical tugging on the cell. Nuclear deformation induced by the stiffening of the ECM may promote gene regulation changes by physically revealing or concealing transcription factor binding sites or through the regulation of key mechanotransducers [19,87]. 

Stiffness can also directly mediate the activity of transcription factors in cancer cells. For example, stiff substratum drives NFκB activation in lung adenocarcinoma cells through actomyosin contractility [87,93]. The effects of ECM stiffening on the nucleus go beyond the regulation of transcription factors. Integrin activation and focal adhesion assembly cause dissociation of protein–protein structures in nuclear Cajal bodies (CB). CBs are involved in RNA processing and splicing and telomere maintenance. Integrin-mediated activation of β-catenin and Myc induces expression of the microRNA miR-18a, which downregulates the tumor suppressor phosphatase and tensin homolog (PTEN). The specific changes in gene expression, signaling pathways, and nuclear morphology that occur in response to mechanical cues from the ECM affect overall cell behavior [88,94,95,96]. Thus, the function of ECM goes beyond just being a scaffold to maintain tissue structure and also plays a role in regulating cell proliferation, differentiation, and migration. 

MSCs cultured on polyacrylamide gels that mimic the brain, muscle, and bone tissue stiffness differentiate into neuronal-like cells, myoblasts, and osteoblasts, respectively, highlighting the importance of ECM stiffness in regulating cell type [97]. Biochemical and mechanical signaling regulate the quiescence of MSCs in the bone marrow. In the same line, by using soluble factors to induce the differentiation of adult neural stem cells, their ultimate fate is significantly influenced by the surrounding microenvironment’s stiffness [87]. Stiff microenvironments can also stimulate signaling through integrin-linked kinase (ILK), leading to cancer stem cell (CSC) –like gene expression [37]. 

Changes in cell behavior stimulated by an increase in stiffness can also affect later stage tumors by initiating EMT or further enhancing proliferation. Similarly, stiffness drives the switch in TGF-β from a tumor suppressor to an EMT inducer [8,87]. Altogether, these studies highlight the importance of mechanical cues from the ECM in modulating cell behavior.

GBM shows that there is a robust cell–ECM interaction at play due to extensive ECM compositional alterations, stiffening, and mechanical stresses [20,98]. Here, the process and mechanism of how GBM cells sense this restructures ECM is defined through a couple of mechanosensors and their signaling pathways. With a focus on the effect of matrix stiffening on the adaptive response of GBM cells, the most important hubs in mechanotransduction are described in the following sections.

### 5.1. F-Actin 

**Figure 5:**
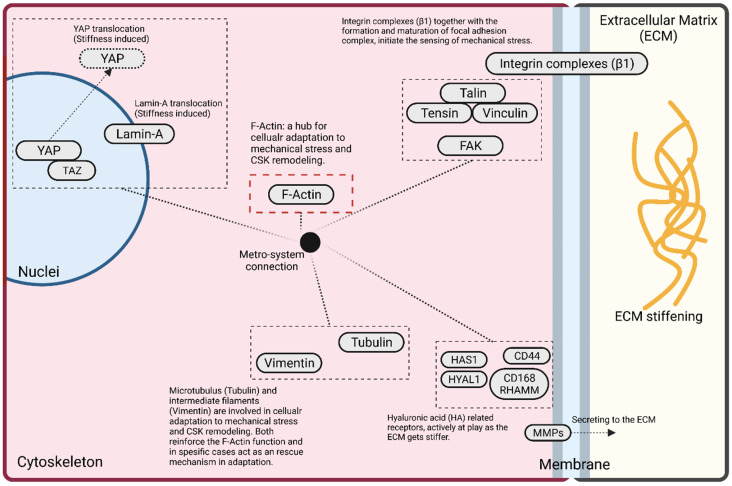
Mechanotransduction in GBM cells. Extracellular matrix (ECM) stiffening applies mechanical stress to GBM cells which activates focal adhesion complex formation. Focal adhesion complexes transmit the mechanical stress to cytoskeleton and initiate the CSK remodeling, where F-actin polymerization is highlighted as the hub which orchestrates a metro-system connection between several adaptive cellular signaling pathways [19,20,101,103,120,121,122].

Actin filaments are the most abundant component of the cell cytosol. Together with tubulin, they help the cell to maintain its structure and move within the matrix [99]. Actin filaments play a key role in numerous signaling pathways as they are connected to many other compartments within the cells and are essential in local motion [100]. The actin legs developed from the cytoskeleton, which facilitate movement, are filopodia. Actin expansion appears in two different types, lamellipodia and filopodia. Filopodia extends the cytoskeleton domain to the surrounding ECM, forcing cells to move forward, and lamellipodia reforms actin expansion towards the cell body itself, which generates a drag flow force that facilitates cell movement [101]. F-actin is known as the hub of mechanotransduction in GBM [50,91,102,103] and is drastically affected by mechanical stress [20]. F-actin can sense the matrix rigidity through integrins connected to focal adhesion sites, where stiffness can intervene to mediate its polymerization (Figure 5).

### 5.2. Focal Adhesion

It is known that the first hallmark of increased ECM stiffness is the stimulation of focal adhesion complex formation in GBM cells [90,104]. Focal adhesion sites are rich in integrin adhesion receptors and play a crucial role in bidirectional transmembrane communication [105]. Focal adhesions are the mechanical linkages to the ECM, which directs the crosstalk of many signaling proteins at the integrin sites [106]. Inside the cell, integrin binds to the cytoskeleton via adapter proteins such as talin, α-actinin, filamin, vinculin, and tensin [94]. In parallel, focal adhesion kinase (FAK) associates with this integrin-adapter protein–cytoskeleton complex to form the basis of the focal adhesion [107]. The dynamic assembly and disassembly of focal adhesions play a central role in cell migration. During migration, both the composition and the morphology of the focal adhesion continuously reorganize. As the cell progresses along a particular path, focal adhesion sites move closer and closer to the cell’s trailing edge [108]. The assembly of nascent focal adhesions is highly dependent on the process of retrograde actin flow [109]. This phenomenon in cell migration occurs where actin filaments polymerize at the leading edge and flow back towards the cell body. This provides the source of traction required for migration and advanced cell movements [102]. The focal adhesion acts as a molecular clutch when it deploys to the ECM and impedes the actin’s retrograde movement, thus generating the pulling (traction) force at the adhesion site, guiding the cell to move forward [110,111].

### 5.3. YAP

YAP (yes-associated protein) is a protein that acts as a transcriptional regulator by activating the transcription of genes involved in cell proliferation and suppressing apoptotic genes. YAP is inhibited in the Hippo signaling pathway, which controls tumor suppression [112]. Additionally, YAP is regulated by mechanical cues such as extracellular matrix (ECM) rigidity, strain, shear stress, and related processes on cytoskeletal integrity [20,92]. YAP localization is strongly mediated by mechanical cues. These mechanically induced localization phenomena are thought to result from nuclear flattening–induced pore size change, mechanosensitive nuclear membrane ion channels, mechanical protein stability, or a wide range of additional factors. The nuclear softening phenotype of cancer cells would promote nuclear flattening in response to a force, causing YAP localization, which could explain its overexpression and promoted proliferation in oncogenic cells [113,114]. Similarly, the opposite effect of nuclear stiffening, due to various stimuli such as an overexpression of lamin-A, has been shown to decrease nuclear YAP localization [115,116]. 

YAP activation is very important in GBM cells’ adaptive response to the ECM stiffening [20]. YAP is activated within the cell cytosol when cells sense a soft ECM, while relocating to the nucleus as the ECM stiffens [115]. As F-actin polymerization sharply increases with ECM stiffening, overexpressed F-actin compresses the nucleus [117], which stimulates YAP translocation [20,92]. As the matrix stiffens, YAP localizes around the nucleus membrane, which is reported to be connected with lamin-A distribution [116]. It is understood that F-actin expression regulates lamin-A distribution, activates YAP within the nucleus, and dictates its localization around the nucleus membrane. Direct evidence showed that YAP localization remodels as cells navigate through confined spaces [118]. On top of a stiff matrix, yet at rest from solid forces, YAP is distributed on the nucleus. Once cells enter through confined channels and sense the solid force, YAP moves beyond the nucleus membrane and enters into cytosol. This translocation helps the cells adapt themselves to the microenvironment to survive. Interestingly, it is reported that GBM cells soften upon navigating through confined spaces, showing that the mechanical stress and solid force soften GBM cells [118,119]. Figure 5 demonstrates the most important players in mechanotransduction signaling and their metro-system connection.

Upon activation of several mechanosensors, the establishment of associated signaling pathways and mediation of cell components, cell cytoskeleton remodeling rises as the hallmark of the adaptive response of GBM cells to ECM compositional alterations, stiffening, and applied mechanical stresses [20,101,120,123]. The remodeling of the cytoskeleton is at play in favor of the invasiveness of GBM and facilitates the migratory state. Within normal ECM, cells are mostly rounded, leading to a healthy cell division. However, with ECM stiffening, cell elongation significantly increases. Elongated cells have expanded cytoskeletons with stretched morphology, which helps them to move, propagate, and proliferate quickly, interrupting default apoptosis and cell division [124].

## 6. Current Bioengineered Strategies: In Vitro Tumor Microenvironment (TME) Models

In vitro bioengineered strategies significantly improved the understanding of the role of mechanical cues and ECM alterations on GBM progression. Numerous hydrogels have been developed to mimic brain ECM properties to perform 3D cell studies where cells can sense the stiffness difference of the gel substrate and start to adapt to it [125]. With the rising field of the physics of cancer, where the focus is mainly on the effect of ECM on cells, routine cell culture platforms such as cell culture plates and flasks cannot be considered as relevant anymore as the experimental setup. Therefore, mimicking the native TME is vital. Hydrogels, a 3D network, provide mechanical properties similar to tissues [126]. Their chemical and mechanical properties are tunable by optimizing their formulation and incorporating different additives [127]. Polymeric gels are a key to developing cell-friendly scaffolds where most of the properties are tunable. Although numerous gels have been developed in recent years, they still lack the ability to mimic the native TME by fully recapitulating its chemical composition and related mechanics. Most of the gels can only mimic some aspects of the ECM, depending on the research question, or not all. Recently, patient-specific materials have emerged into the field, making it possible to push the boundaries and become closer to native brain ECM [127,128]. We provide the five most common, highly biocompatible, and easily tunable materials to develop gels for GBM cell studies, along with the references of the respected studies for further details that go beyond the scope of the this review (Table 1).

## 7. Future Therapeutics: From Understanding to Tackling

Because of its separation from the surrounding systems and the existence of the blood–brain barrier (BBB), which impedes the movement of many immune cells and chemotherapeutics, GBM imposes a big challenge for drug delivery [5,66]. Moreover, for drug development, the complex genetic and molecular environment of glioblastoma is an obstacle, which led to the lack of drug approval in the past decade. Maximum surgical resection of the tumor with concomitant chemoradiation using the alkylating agent temozolomide (TMZ) and accompanied by the adjuvant TMZ for a total of 6 months is the highlight of glioblastoma treatment. The addition of the tumor treating field (TTF) to the existing standard of care, which is a system worn by the patient on the scalp, is another treatment modality that operates by providing alternating electrical fields that destroy the microtubules in the mitotic spindle leading to the death of the tumor cell. However, considering the poor compliance rates of its usage and high cost, it is not considered a functional and suitable therapy for patients [155,156]. The present review highlighted the effect of physical traits of cancer, specifically ECM stiffening, on GBM rapid progression. It is understood that ECM alteration is one of the keys to drive tumor progression and GBM cell invasiveness. Thus, new strategies to tackle the GBM growth are based on controlling and limiting ECM alterations and their consequences. In vitro tumor models based on hydrogels made it possible to test and screen many inhibitors. Koh et al. [124] used a patient-specific hydrogel to test the HA related inhibitors. With encapsulating an MMPs inhibitor (SB-3CT) and HAS inhibitor (4-MU) separately within the hydrogel, they showed that inhibiting both MMPs and HA synthases genes (HAS) significantly suppressed GBM cell motility and migration, while cell elongation was also decreased. Razinia et al. [157] showed how stiffness-dependent GBM cell motility is uncoupled by deletion of CD44. By inhibiting CD44, GBM cells are less sensitive to the ECM alterations, mostly HA expression, and therefore the cell migration is limited largely. By driving cell transformation and gene expression changes, stiffness can help confer a survival advantage to cancer cells. For example, the morphology and proliferation of cancer cells can actually become insensitive to ECM stiffness through regulation of caveolin-1 (Cav1), a scaffold protein essential for integrin-mediated mechanotransduction [87]. Insensitivity to stiffness can enhance cancer cells’ ability to thrive in vivo [158]. These findings show that restraining ECM alterations results in a successful outcome to control GBM tumor progression, suggesting that inhibitors are potential winners over drugs such as TMZ.

## 8. Conclusions

The physics of cancer renders new insight into GBM tumor development and progression. Drastic ECM alterations are the birthplace of most physical traits of cancer where ECM stiffening and generation of mechanical stresses are marked as the most important physical stimuli in cancer progression. As the ECM stiffens, it fuels tumor progression with the acceleration of GBM cell’s proliferation, migration, and elongation. In fact, ECM stiffening activates a cascade of events, including several intercellular and intracellular signaling pathways as part of the mechanotransduction process to initiate the adaptive response of cells to the altered TME and whole brain ECM at later stages. The principal and newly discovered pathways involved in cell–ECM interactions are highlighted in this review. Although most of these signaling pathways are not explored thoroughly, they offer a more profound understanding of GBM cellular properties and characteristics. Hydrogels have emerged into the field as reliable in vitro models for cell studies by recapitulating some native properties of the brain’s ECM. By gaining a better understanding of these concepts, future therapeutics can be developed to more effectively target GBM tumor invasiveness. 

## Figures and Tables

**Table 1:** The five widely used materials as hydrogels to mimic the glioblastoma tumor microenvironment for cell studies. Advantages and limitations of each material are highlighted.

Material	Advantages	Disadvantages
Hyaluronic Acid (HA) [44,62,129,130,131,132]	- Easily tunable in properties with playing with HA molecular weight (Mw) - Mimicking ECM stiffening with varying the HA concentration or density of crosslinking - Mimicking the HA over-excess within the brain to study HA-related cell signaling pathways	- Complex chemistry for crosslinking (chemical modifications such as methacrylation is required) - Low cell adhesion properties - High degradation over time - Unstable structure
Collagen [16,39,43,64,104,126,133,134,135]	- Easy gelation with thermal crosslinking - Easily tunable properties with chemical modification (methacrylation) - Mimicking ECM stiffening in a wide range - Stable structure with low degradation (Suitable for long time cell culture) - Fully transparent (high resolution imaging) - Close mimic of tissue mechanics	- Aligned nano-topography from its fibrous structure might promote cell migration as a false readout - Poor mimic of native brain chemical composition
Gelatin–Methacrylate (GelMa) [44,62,115,136,137,138,139,140,141]	- Easily tunable in stiffness - Close mimic of tissue mechanics. - Transparent (high resolution imaging) - Suitable to use as a bioink for bioprinting - High stability with low degradation	- Complex chemistry for gel preparation - Not optimal crosslinking degree - Poor mimic of native brain chemical composition - Not compatible with several polymers and additives - Presence of free ions
Brain Decellularized Tissue (dECM) [124,127,142,143,144,145,146,147,148,149]	- Close mimic of the brain chemical composition with native properties - High cell adhesion properties - Compatible with many types of polymers to tune the properties and stiffness - Close mimic for tissue microarchitecture - Suitable scaffold for drug screening purposes	- Complex chemistry for gel preparation - Decellularization process can damage proteins (depending on the procedure) - Not transparent (imaging challenges) - High degradation
Human Blood Plasma [39,43,127,150,151,152,153,154]	- High cell adhesion properties - Compatible with many types of polymers to tune the properties and stiffness - Including growth factors and native tissue components - Suitable scaffold for drug screening	- Cannot mimic the mechanics of the tissue - Fast degradation - Not transparent (imaging challenges) - Complex chemistry - Heterogeneous crosslinking

## Data Availability

Not applicable.

## References

[1] Ohgaki H., Kleihues P. (2013). The Definition of Primary and Secondary Glioblastoma. Clin. Cancer Res..

[2] Stathis A. (2016). Treatment Overview. Handb. Lymphoma.

[3] Preusser M., De Ribaupierre S., Wöhrer A., Erridge S.C., Hegi M., Weller M., Stupp R. (2011). Current Concepts and Management of Glioblastoma. Ann. Neurol..

[4] Bastiancich C., Danhier P., Préat V., Danhier F. (2016). Anticancer Drug-Loaded Hydrogels as Drug Delivery Systems for the Local Treatment of Glioblastoma. J. Control. Release.

[5] Khandwala K., Mubarak F., Minhas K. (2020). The Many Faces of Glioblastoma: Pictorial Review of Atypical Imaging Features. Neuroradiol. J..

[6] Vollmann-Zwerenz A., Leidgens V., Feliciello G., Klein C.A., Hau P. (2020). Tumor Cell Invasion in Glioblastoma. Int. J. Mol. Sci..

[7] Virga J., Szivos L., Hortobágyi T., Chalsaraei M.K., Zahuczky G., Steiner L., Tóth J., Reményi-Puskár J., Bognár L., Klekner A. (2019). Extracellular Matrix Differences in Glioblastoma Patients with Different Prognoses. Oncol. Lett..

[8] Rejniak K.A. (2016). Systems Biology of Tumor Microenvironment: Quantitative Modeling and Simulations.

[9] Neftel C., Laffy J., Filbin M.G., Hara T., Shore M.E., Rahme G.J., Richman A.R., Silverbush D., Shaw M.L., Hebert C.M. (2019). An Integrative Model of Cellular States, Plasticity, and Genetics for Glioblastoma. Cell.

[10] Mouw J.K., Ou G., Weaver V.M. (2014). Extracellular Matrix Assembly: A Multiscale Deconstruction. Nat. Rev. Mol. Cell Biol..

[11] Restall I., Bozek D.A., Chesnelong C., Weiss S., Luchman H.A. (2018). Live-Cell Imaging Assays to Study Glioblastoma Brain Tumor Stem Cell Migration and Invasion. J. Vis. Exp..

[12] Wolf K.J., Chen J., Coombes J.D., Aghi M.K., Kumar S. (2019). Dissecting and Rebuilding the Glioblastoma Microenvironment with Engineered Materials. Nat. Rev. Mater..

[13] Budday S., Ovaert T.C., Holzapfel G.A., Steinmann P., Kuhl E. (2019). Fifty Shades of Brain: A Review on the Mechanical Testing and Modeling of Brain Tissue.

[14] Grundy T.J., De Leon E., Griffin K.R., Stringer B.W., Day B.W., Fabry B., Cooper-White J., O’Neill G.M. (2016). Differential Response of Patient-Derived Primary Glioblastoma Cells to Environmental Stiffness. Sci. Rep..

[15] Nia H.T., Munn L.L., Jain R.K. (2019). Mapping Physical Tumor Microenvironment and Drug Delivery. Clin. Cancer Res..

[16] Palamà I.E., D’Amone S., Cortese B. (2018). Microenvironmental Rigidity of 3D Scaffolds and Influence on Glioblastoma Cells: A Biomaterial Design Perspective. Front. Bioeng. Biotechnol..

[17] Mair D.B., Ames H.M., Li R. (2018). Mechanisms of Invasion and Motility of High-Grade Gliomas in the Brain. Mol. Biol. Cell.

[18] Wirtz D., Konstantopoulos K., Searson P.C. (2011). The Physics of Cancer: The Role of Physical Interactions and Mechanical Forces in Metastasis. Nat. Rev. Cancer.

[19] Martino F., Perestrelo A.R., Vinarský V., Pagliari S., Forte G. (2018). Cellular Mechanotransduction: From Tension to Function. Front. Physiol..

[20] Nia H.T., Munn L.L., Jain R.K. (2020). Physical Traits of Cancer. Science.

[21] Nagelkerke A., Bussink J., Rowan A.E., Span P.N. (2015). The Mechanical Microenvironment in Cancer: How Physics Affects Tumours. Semin. Cancer Biol..

[22] Pogoda K., Chin L., Georges P.C., Byfield F.J., Bucki R., Kim R., Weaver M., Wells R.G., Marcinkiewicz C., Janmey P.A. (2014). Compression Stiffening of Brain and Its Effect on Mechanosensing by Glioma Cells. New J. Phys..

[23] Zhang Q., Yu Y., Zhao H. (2016). The Effect of Matrix Stiffness on Biomechanical Properties of Chondrocytes. Acta Biochim. Biophys. Sin..

[24] Hubmacher D., Apte S.S. (2013). The Biology of the Extracellular Matrix: Novel Insights. Curr. Opin. Rheumatol..

[25] Quail D.F., Joyce J.A. (2017). The Microenvironmental Landscape of Brain Tumors. Cancer Cell.

[26] Gandhi N.S., Mancera R.L. (2008). The Structure of Glycosaminoglycans and Their Interactions with Proteins. Chem. Biol. Drug Des..

[27] Ruoslahti E. (1988). Structural and Biology. Cell Differ..

[28] Iozzo R.V. (1998). Matrix Proteoglycans: From Molecular Design to Cellular Function. Annu. Rev. Biochem..

[29] Miyata S., Kitagawa H. (2016). Chondroitin 6-Sulfation Regulates Perineuronal Net Formation by Controlling the Stability of Aggrecan. Neural Plast..

[30] Teodori L., Costa A., Marzio R., Perniconi B., Coletti D., Adamo S., Gupta B., Tarnok A. (2014). Native Extracellular Matrix: A New Scaffolding Platform for Repair of Damaged Muscle. Front. Physiol..

[31] Shoulders M.D., Raines R.T. (2009). Collagen Structure and Stability Ann Rev Biochemistry. Annu. Rev. Biochem..

[32] Payne L.S., Huang P.H. (2013). The Pathobiology of Collagens in Glioma. Mol. Cancer Res..

[33] Sood D., Chwalek K., Stuntz E., Pouli D., Du C., Tang-Schomer M., Georgakoudi I., Black L.D., Kaplan D.L. (2016). Fetal Brain Extracellular Matrix Boosts Neuronal Network Formation in 3D Bioengineered Model of Cortical Brain Tissue. ACS Biomater. Sci. Eng..

[34] Debelle L., Tamburro A.M. (1999). Elastin: Molecular Description and Function. Int. J. Biochem. Cell Biol..

[35] Singh P., Carraher C., Schwarzbauer J.E. (2010). Assembly of Fibronectin Extracellular Matrix. Annu. Rev. Cell Dev. Biol..

[36] Johansson S., Svineng G., Wennerberg K., Armulik A., Lohikangas L. (1997). Fibronectin-Integrin Interactions. Front. Biosci..

[37] Brizzi M.F., Tarone G., Defilippi P. (2012). Extracellular Matrix, Integrins, and Growth Factors as Tailors of the Stem Cell Niche. Curr. Opin. Cell Biol..

[38] Belousov A., Titov S., Shved N., Garbuz M., Malykin G., Gulaia V., Kagansky A., Kumeiko V. (2019). The Extracellular Matrix and Biocompatible Materials in Glioblastoma Treatment. Front. Bioeng. Biotechnol..

[39] Heffernan J.M., Sirianni R.W. (2018). Modeling Microenvironmental Regulation of Glioblastoma Stem Cells: A Biomaterials Perspective. Front. Mater..

[40] Christofori G., Semb H. (1999). The Role of the Cell-Adhesion Molecule E-Cadherin as a Tumour-Suppressor Gene. Trends Biochem. Sci..

[41] Elangbam C.S., Qualls C.W., Dahlgren R.R. (1997). Cell Adhesion Molecules—Update. Vet. Pathol..

[42] Lukashev M.E., Werb Z. (1998). ECM Signalling: Orchestrating Cell Behaviour and Misbehaviour. Trends Cell Biol..

[43] Cha J., Kim P. (2017). Biomimetic Strategies for the Glioblastoma Microenvironment. Front. Mater..

[44] Chen J.W.E., Pedron S., Shyu P., Hu Y., Sarkaria J.N., Harley B.A.C. (2018). Influence of Hyaluronic Acid Transitions in Tumor Microenvironment on Glioblastoma Malignancy and Invasive Behavior. Front. Mater..

[45] Riegler J., Labyed Y., Rosenzweig S., Javinal V., Castiglioni A., Dominguez C.X., Long J.E., Li Q., Sandoval W., Junttila M.R. (2018). Tumor Elastography and Its Association with Collagen and the Tumor Microenvironment. Clin. Cancer Res..

[46] Lin T.C., Yang C.H., Cheng L.H., Chang W.T., Lin Y.R., Cheng H.C. (2019). Fibronectin in Cancer: Friend or Foe. Cells.

[47] De Oliveira Rosario L.V., Da Rosa B.G., Goncalves T.L., Matias D.I.L., Freitas C., Ferrer V.P. (2020). Glioblastoma Factors Increase the Migration of Human Brain Endothelial Cells in Vitro by Increasing MMP-9/CXCR4 Levels. Anticancer Res..

[48] Barnes J.M., Przybyla L., Weaver V.M. (2017). Tissue Mechanics Regulate Brain Development, Homeostasis and Disease. J. Cell Sci..

[49] Stewart D.C., Rubiano A., Dyson K., Simmons C.S. (2017). Mechanical Characterization of Human Brain Tumors from Patients and Comparison to Potential Surgical Phantoms. PLoS ONE.

[50] Li X., Wang J. (2020). Mechanical Tumor Microenvironment and Transduction: Cytoskeleton Mediates Cancer Cell Invasion and Metastasis. Int. J. Biol. Sci..

[51] Duan Q., Zhang H., Zheng J., Zhang L. (2020). Turning Cold into Hot: Firing up the Tumor Microenvironment. Trends Cancer.

[52] Necas J., Bartosikova L., Brauner P., Kolar J. (2008). Hyaluronic Acid (Hyaluronan): A Review. Vet. Med..

[53] Khaing Z.Z., Seidlits S.K. (2015). Hyaluronic Acid and Neural Stem Cells: Implications for Biomaterial Design. J. Mater. Chem. B.

[54] Delpech B., Maingonnat C., Girard N., Chauzy C., Olivier A., Maunoury R., Tayot J., Creissard P. (1993). Hyaluronan and Hyaluronectin in the Extracellular Matrix of Human Brain Tumour Stroma. Eur. J. Cancer.

[55] Zimmermann D.R., Dours-Zimmermann M.T. (2008). Extracellular Matrix of the Central Nervous System: From Neglect to Challenge. Histochem. Cell Biol..

[56] Cha J., Kang S.G., Kim P. (2016). Strategies of Mesenchymal Invasion of Patient-Derived Brain Tumors: Microenvironmental Adaptation. Sci. Rep..

[57] Wolf K.J., Kumar S. (2019). Hyaluronic Acid: Incorporating the Bio into the Material. ACS Biomater. Sci. Eng..

[58] Bignami A., Hosley M., Dahl D. (1993). Hyaluronic Acid and Hyaluronic Acid-Binding Proteins in Brain Extracellular Matrix. Anat. Embryol..

[59] Pibuel M.A., Díaz M., Molinari Y., Poodts D., Silvestroff L., Lompardía S.L., Franco P., Hajos S.E. (2020). 4-Methylumbelliferone as a Potent and Selective Antitumor Drug on a Glioblastoma Model. Glycobiology.

[60] Liu M., Tolg C., Turley E. (2019). Dissecting the Dual Nature of Hyaluronan in the Tumor Microenvironment. Front. Immunol..

[61] Kim Y., Kumar S. (2014). CD44-Mediated Adhesion to Hyaluronic Acid Contributes to Mechanosensing and Invasive Motility. Mol. Cancer Res..

[62] Chen J.W.E., Pedron S., Harley B.A.C. (2017). The Combined Influence of Hydrogel Stiffness and Matrix-Bound Hyaluronic Acid Content on Glioblastoma Invasion. Macromol. Biosci..

[63] Erickson A.E., Lan Levengood S.K., Sun J., Chang F.C., Zhang M. (2018). Fabrication and Characterization of Chitosan–Hyaluronic Acid Scaffolds with Varying Stiffness for Glioblastoma Cell Culture. Adv. Healthc. Mater..

[64] Lou J., Stowers R., Nam S., Xia Y., Chaudhuri O. (2018). Stress Relaxing Hyaluronic Acid-Collagen Hydrogels Promote Cell Spreading, Fiber Remodeling, and Focal Adhesion Formation in 3D Cell Culture. Biomaterials.

[65] Zamboni F., Keays M., Hayes S., Albadarin A.B., Walker G.M., Kiely P.A., Collins M.N. (2017). Enhanced Cell Viability in Hyaluronic Acid Coated Poly(Lactic-Co-Glycolic Acid) Porous Scaffolds within Microfluidic Channels. Int. J. Pharm..

[66] Urbanska K., Sokolowska J., Szmidt M., Sysa P. (2014). Glioblastoma Multiforme—An Overview. Wspolczesna Onkol..

[67] Sorribes I.C., Moore M.N.J., Byrne H.M., Jain H.V. (2019). A Biomechanical Model of Tumor-Induced Intracranial Pressure and Edema in Brain Tissue. Biophys. J..

[68] Reetz K., Abbas Z., Costa A.S., Gras V., Tiffin-Richards F., Mirzazade S., Holschbach B., Frank R.D., Vassiliadou A., Krüger T. (2015). Increased Cerebral Water Content in Hemodialysis Patients. PLoS ONE.

[69] Olson J.E., Mishler L., Dimlich R.V.W. (1990). Brain Water Content, Brain Blood Volume, Blood Chemistry, and Pathology in a Model of Cerebral Edema. Ann. Emerg. Med..

[70] Streitberger K.J., Lilaj L., Schrank F., Braun J., Hoffmann K.T., Reiss-Zimmermann M., Käs J.A., Sack I. (2020). How Tissue Fluidity Influences Brain Tumor Progression. Proc. Natl. Acad. Sci. USA.

[71] Schiller J., Huster D. (2012). New Methods to Study the Composition and Structure of the Extracellular Matrix in Natural and Bioengineered Tissues. Biomatter.

[72] Venkatesan R., Lin W., Gurleyik K., He Y.Y., Paczynski R.P., Powers W.J., Hsu C.Y. (2000). Absolute Measurements of Water Content Using Magnetic Resonance Imaging: Preliminary Findings in an in Vivo Focal Ischemic Rat Model. Magn. Reson. Med..

[73] Ciasca G., Sassun T.E., Minelli E., Antonelli M., Papi M., Santoro A., Giangaspero F., Delfini R., De Spirito M. (2016). Nano-Mechanical Signature of Brain Tumours. Nanoscale.

[74] Bunevicius A., Schregel K., Sinkus R., Golby A., Patz S. (2020). REVIEW: MR Elastography of Brain Tumors. NeuroImage Clin..

[75] Fattahi N., Arani A., Perry A., Meyer F., Manduca A., Glaser K., Senjem M.L., Ehman R.L., Huston J. (2016). MR Elastography Demonstrates Increased Brain Stiffness in Normal Pressure Hydrocephalus. Am. J. Neuroradiol..

[76] Maher E.A., Bachoo R.M. (2014). Glioblastoma. https://www.sciencedirect.com/science/article/pii/B9780124105294000784?via%3Dihub.

[77] Bilston L.E. (2018). Soft Tissue Rheology and Its Implications for Elastography: Challenges and Opportunities. NMR Biomed..

[78] Guimarães C.F., Gasperini L., Marques A.P., Reis R.L. (2020). The Stiffness of Living Tissues and Its Implications for Tissue Engineering. Nat. Rev. Mater..

[79] Saleh A., Marhuenda E., Fabre C., Hassani Z., de Weille J., Boukhaddaoui H., Guelfi S., Maldonado I.L., Hugnot J.P., Duffau H. (2019). A Novel 3D Nanofibre Scaffold Conserves the Plasticity of Glioblastoma Stem Cell Invasion by Regulating Galectin-3 and Integrin-Β1 Expression. Sci. Rep..

[80] Sharma P., Sheets K., Elankumaran S., Nain A.S. (2013). The Mechanistic Influence of Aligned Nanofibers on Cell Shape, Migration and Blebbing Dynamics of Glioma Cells. Integr. Biol..

[81] Beliveau A., Thomas G., Gong J., Wen Q., Jain A. (2016). Aligned Nanotopography Promotes a Migratory State in Glioblastoma Multiforme Tumor Cells. Sci. Rep..

[82] Babu P.K.V., Radmacher M. (2019). Mechanics of Brain Tissues Studied by Atomic Force Microscopy: A Perspective. Front. Neurosci..

[83] Palombo F., Winlove C.P., Edginton R.S., Green E., Stone N., Caponi S., Madami M., Fioretto D. (2014). Biomechanics of Fibrous Proteins of the Extracellular Matrix Studied by Brillouin Scattering. J. R. Soc. Interface.

[84] Nia H.T., Datta M., Seano G., Zhang S., Ho W.W., Roberge S., Huang P., Munn L.L., Jain R.K. (2020). In Vivo Compression and Imaging in Mouse Brain to Measure the Effects of Solid Stress. Nat. Protoc..

[85] Munn L.L., Nia H.T. (2019). Mechanosensing Tensile Solid Stresses. Proc. Natl. Acad. Sci. USA.

[86] Seano G., Nia H.T., Emblem K.E., Datta M., Ren J., Krishnan S., Kloepper J., Pinho M.C., Ho W.W., Ghosh M. (2019). Solid Stress in Brain Tumours Causes Neuronal Loss and Neurological Dysfunction and Can Be Reversed by Lithium. Nat. Biomed. Eng..

[87] Simi A.K., Pang M., Nelson C.M. (2018). Extracellular Matrix Stiffness Exists in a Feedback Loop That Drives Tumor Progression. Adv. Exp. Med. Biol..

[88] Huang H., Kamm R.D., Lee R.T. (2004). Cell Mechanics and Mechanotransduction: Pathways, Probes, and Physiology. Am. J. Physiol.-Cell Physiol..

[89] Interests C.F. (1926). News and Views. Nature.

[90] Gkretsi V., Stylianopoulos T. (2018). Cell Adhesion and Matrix Stiffness: Coordinating Cancer Cell Invasion and Metastasis. Front. Oncol..

[91] Provenzano P.P., Keely P.J. (2011). Mechanical Signaling through the Cytoskeleton Regulates Cell Proliferation by Coordinated Focal Adhesion and Rho GTPase Signaling. J. Cell Sci..

[92] Dupont S., Morsut L., Aragona M., Enzo E., Giulitti S., Cordenonsi M., Zanconato F., Le Digabel J., Forcato M., Bicciato S. (2011). Role of YAP/TAZ in Mechanotransduction. Nature.

[93] Gill B.J., Gibbons D.L., Roudsari L.C., Saik J.E., Rizvi Z.H., Roybal J.D., Kurie J.M., West J.L. (2012). A Synthetic Matrix with Independently Tunable Biochemistry and Mechanical Properties to Study Epithelial Morphogenesis and EMT in a Lung Adenocarcinoma Model. Cancer Res..

[94] Wolfenson H., Yang B., Sheetz M.P. (2019). Steps in Mechanotransduction Pathways That Control Cell Morphology. Annu. Rev. Physiol..

[95] Jaalouk D.E., Lammerding J. (2009). Mechanotransduction Gone Awry. Nat. Rev. Mol. Cell Biol..

[96] Farge E. (2011). Mechanotransduction in Development.

[97] Sun M., Chi G., Li P., Lv S., Xu J., Xu Z., Xia Y., Tan Y., Xu J., Li L. (2018). Effects of Matrix Stiffness on the Morphology, Adhesion, Proliferation and Osteogenic Differentiation of Mesenchymal Stem Cells. Int. J. Med. Sci..

[98] Kalinin V. (2020). Cell–Extracellular Matrix Interaction in Glioma Growth. In Silico Model. J. Integr. Bioinform..

[99] Dominguez R., Holmes K.C. (2011). Actin Structure and Function. Annu. Rev. Biophys..

[100] Pellegrin S., Mellor H. (2007). Actin Stress Fibers. J. Cell Sci..

[101] Seetharaman S., Etienne-Manneville S. (2020). Cytoskeletal Crosstalk in Cell Migration. Trends Cell Biol..

[102] Schwarz U.S., Gardel M.L. (2012). United We Stand-Integrating the Actin Cytoskeleton and Cell-Matrix Adhesions in Cellular Mechanotransduction. J. Cell Sci..

[103] Bhadriraju K., Hansen L.K. (2002). Extracellular Matrix- and Cytoskeleton-Dependent Changes in Cell Shape and Stiffness. Exp. Cell Res..

[104] Schlunck G., Han H., Wecker T., Kampik D., Meyer-ter-Vehn T., Grehn F. (2008). Substrate Rigidity Modulates Cell-Matrix Interactions and Protein Expression in Human Trabecular Meshwork Cells. Investig. Ophthalmol. Vis. Sci..

[105] Giardiello F.M., Hylind L.M., Trimbath J.D., Hamilton S.R., Romans K.E., Cruz-Correa M., Corretti M.C., Offerhaus G.J.A., Yang V.W. (2005). Oral Contraceptives and Polyp Regression in Familial Adenomatous Polyposis. Gastroenterology.

[106] Wozniak M.A., Modzelewska K., Kwong L., Keely P.J. (2004). Focal Adhesion Regulation of Cell Behavior. Biochim. Biophys. Acta-Mol. Cell Res..

[107] Schlaepfer D.D., Hauck C.R., Sieg D.J. (1999). Signaling through Focal Adhesion Kinase. Prog. Biophys. Mol. Biol..

[108] Janiszewska M., Primi M.C., Izard T. (2020). Cell Adhesion in Cancer: Beyond the Migration of Single Cells. J. Biol. Chem..

[109] Alexandrova A.Y., Arnold K., Schaub S., Vasiliev J.M., Meister J.J., Bershadsky A.D., Verkhovsky A.B. (2008). Comparative Dynamics of Retrograde Actin Flow and Focal Adhesions: Formation of Nascent Adhesions Triggers Transition from Fast to Slow Flow. PLoS ONE.

[110] Kanoldt V., Kluger C., Barz C., Schweizer A.L., Ramanujam D., Windgasse L., Engelhardt S., Chrostek-Grashoff A., Grashoff C. (2020). Metavinculin Modulates Force Transduction in Cell Adhesion Sites. Nat. Commun..

[111] Umesh V., Rape A.D., Ulrich T.A., Kumar S. (2014). Microenvironmental Stiffness Enhances Glioma Cell Proliferation by Stimulating Epidermal Growth Factor Receptor Signaling. PLoS ONE.

[112] Abylkassov R., Xie Y. (2016). Role of Yes-Associated Protein in Cancer: An Update (Review). Oncol. Lett..

[113] Caliari S.R., Perepelyuk M., Cosgrove B.D., Tsai S.J., Lee G.Y., Mauck R.L., Wells R.G., Burdick J.A. (2016). Stiffening Hydrogels for Investigating the Dynamics of Hepatic Stellate Cell Mechanotransduction during Myofibroblast Activation. Sci. Rep..

[114] Bandaru P., Cefaloni G., Vajhadin F., Lee K.J., Kim H.J., Cho H.J., Hartel M.C., Zhang S., Sun W., Goudie M.J. (2020). Mechanical Cues Regulating Proangiogenic Potential of Human Mesenchymal Stem Cells through YAP-Mediated Mechanosensing. Small.

[115] Vallejo-Giraldo C., Genta M., Cauvi O., Goding J., Green R. (2020). Hydrogels for 3D Neural Tissue Models: Understanding Cell-Material Interactions at a Molecular Level. Front. Bioeng. Biotechnol..

[116] Koushki N., Ghagre A., Srivastava L.K., Sitaras C., Yoshie H., Molter C., Ehrlicher A. (2020). Lamin A Redistribution Mediated by Nuclear Deformation Determines Dynamic Localization of YAP. BioRxiv.

[117] Belaadi N., Aureille J., Guilluy C. (2016). Under Pressure: Mechanical Stress Management in the Nucleus. Cells.

[118] Rianna C., Radmacher M., Kumar S. (2020). Direct Evidence That Tumor Cells Soften When Navigating Confined Spaces. Mol. Biol. Cell.

[119] Paul C.D., Mistriotis P., Konstantopoulos K. (2017). Cancer Cell Motility: Lessons from Migration in Confined Spaces. Nat. Rev. Cancer.

[120] Shen K., Kenche H., Zhao H., Li J., Stone J. (2019). The Role of Extracellular Matrix Stiffness in Regulating Cytoskeletal Remodeling via Vinculin in Synthetic Smooth Muscle Cells. Biochem. Biophys. Res. Commun..

[121] Pegoraro A.F., Janmey P., Weitz D.A. (2017). Mechanical Properties of the Cytoskeleton and Cells. Cold Spring Harb. Perspect. Biol..

[122] Harburger D.S., Calderwood D.A. (2009). Integrin Signalling at a Glance. J. Cell Sci..

[123] Janmey P.A. (1998). The Cytoskeleton and Cell Signaling: Component Localization and Mechanical Coupling. Physiol. Rev..

[124] Koh I., Cha J., Park J., Choi J., Kang S.G., Kim P. (2018). The Mode and Dynamics of Glioblastoma Cell Invasion into a Decellularized Tissue-Derived Extracellular Matrix-Based Three-Dimensional Tumor Model. Sci. Rep..

[125] Discher D.E., Janmey P., Wang Y.L. (2005). Tissue Cells Feel and Respond to the Stiffness of Their Substrate. Science.

[126] Caló E., Khutoryanskiy V.V. (2015). Biomedical Applications of Hydrogels: A Review of Patents and Commercial Products. Eur. Polym. J..

[127] Raman R., Langer R. (2019). Biohybrid Design Gets Personal: New Materials for Patient-Specific Therapy. Adv. Mater..

[128] Gomez-Roman N., Chalmers A.J. (2019). Patient-Specific 3D-Printed Glioblastomas. Nat. Biomed. Eng..

[129] Pogoda K., Bucki R., Byfield F.J., Cruz K., Lee T., Marcinkiewicz C., Janmey P.A. (2017). Soft Substrates Containing Hyaluronan Mimic the Effects of Increased Stiffness on Morphology, Motility, and Proliferation of Glioma Cells. Biomacromolecules.

[130] Karabiyik Acar O., Kayitmazer A.B., Torun Kose G. (2018). Hyaluronic Acid/Chitosan Coacervate-Based Scaffolds. Biomacromolecules.

[131] Burdick J.A., Prestwich G.D. (2011). Hyaluronic Acid Hydrogels for Biomedical Applications. Adv. Mater..

[132] Khunmanee S., Jeong Y., Park H. (2017). Crosslinking Method of Hyaluronic-Based Hydrogel for Biomedical Applications. J. Tissue Eng..

[133] Sung K.E., Su G., Pehlke C., Trier S.M., Eliceiri K.W., Keely P.J., Friedl A., Beebe D.J. (2009). Control of 3-Dimensional Collagen Matrix Polymerization for Reproducible Human Mammary Fibroblast Cell Culture in Microfluidic Devices. Biomaterials.

[134] Rao S.S., Dejesus J., Short A.R., Otero J.J., Sarkar A., Winter J.O. (2013). Glioblastoma Behaviors in Three-Dimensional Collagen-Hyaluronan Composite Hydrogels. ACS Appl. Mater. Interfaces.

[135] Ayuso J.M., Monge R., Martínez-González A., Virumbrales-Muñoz M., Llamazares G.A., Berganzo J., Hernández-Laín A., Santolaria J., Doblaré M., Hubert C. (2017). Glioblastoma on a Microfluidic Chip: Generating Pseudopalisades and Enhancing Aggressiveness through Blood Vessel Obstruction Events. Neuro-Oncology.

[136] Sakthivel K., Kumar H., Mohamed M.G.A., Talebjedi B., Shim J., Najjaran H., Hoorfar M., Kim K. (2020). High Throughput Screening of Cell Mechanical Response Using a Stretchable 3D Cellular Microarray Platform. Small.

[137] Mora-Boza A., Włodarczyk-Biegun M.K., Del Campo A., Vázquez-Lasa B., Román J.S. (2020). Glycerylphytate as an Ionic Crosslinker for 3D Printing of Multi-Layered Scaffolds with Improved Shape Fidelity and Biological Features. Biomater. Sci..

[138] Xu L., Wang C., Cui Y., Li A., Qiao Y., Qiu D. (2019). Conjoined-Network Rendered Stiff and Tough Hydrogels from Biogenic Molecules. Sci. Adv..

[139] Jiang T., Munguia-Lopez J.G., Gu K., Bavoux M.M., Flores-Torres S., Kort-Mascort J., Grant J., Vijayakumar S., De Leon-Rodriguez A., Ehrlicher A.J. (2020). Engineering Bioprintable Alginate/Gelatin Composite Hydrogels with Tunable Mechanical and Cell Adhesive Properties to Modulate Tumor Spheroid Growth Kinetics. Biofabrication.

[140] Bahram M., Mohseni N., Moghtader M. (2016). An Introduction to Hydrogels and Some Recent Applications. Emerg. Concepts Anal. Appl. Hydrogels.

[141] Liaw C.Y., Ji S., Guvendiren M. (2018). Engineering 3D Hydrogels for Personalized In Vitro Human Tissue Models. Adv. Healthc. Mater..

[142] Shin Y.J., Shafranek R.T., Tsui J.H., Walcott J., Nelson A., Kim D.H. (2020). 3D Bioprinting of Mechanically Tuned Bioinks Derived from Cardiac Decellularized Extracellular Matrix. Acta Biomater..

[143] Choudhury D., Tun H.W., Wang T., Naing M.W. (2018). Organ-Derived Decellularized Extracellular Matrix: A Game Changer for Bioink Manufacturing?. Trends Biotechnol..

[144] Ferreira L.P., Gaspar V.M., Mano J.F. (2020). Decellularized Extracellular Matrix for Bioengineering Physiomimetic 3D in Vitro Tumor Models. Trends Biotechnol..

[145] Kabirian F., Mozafari M. (2020). Decellularized ECM-Derived Bioinks: Prospects for the Future. Methods.

[146] Seo Y., Jeong S., Chung J.J., Kim S.H., Choi N., Jung Y. (2020). Development of an Anisotropically Organized Brain DECM Hydrogel-Based 3D Neuronal Culture Platform for Recapitulating the Brain Microenvironment in Vivo. ACS Biomater. Sci. Eng..

[147] Lam D., Enright H.A., Cadena J., Peters S.K.G., Sales A.P., Osburn J.J., Soscia D.A., Kulp K.S., Wheeler E.K., Fischer N.O. (2019). Tissue-Specific Extracellular Matrix Accelerates the Formation of Neural Networks and Communities in a Neuron-Glia Co-Culture on a Multi-Electrode Array. Sci. Rep..

[148] Sackett S.D., Tremmel D.M., Ma F., Feeney A.K., Maguire R.M., Brown M.E., Zhou Y., Li X., O’Brien C., Li L. (2018). Extracellular Matrix Scaffold and Hydrogel Derived from Decellularized and Delipidized Human Pancreas. Sci. Rep..

[149] Simsa R., Rothenbücher T., Gürbüz H., Ghosheh N., Emneus J., Jenndahl L., Kaplan D.L., Bergh N., Serrano A.M., Fogelstrand P. (2021). Brain Organoid Formation on Decellularized Porcine Brain ECM Hydrogels. PLoS ONE.

[150] Malagón-Romero D., Hernández N., Cardozo C., Godoy-Silva R.D. (2014). Rheological Characterization of a Gel Produced Using Human Blood Plasma and Alginate Mixtures. J. Mech. Behav. Biomed. Mater..

[151] Tamayol A., Najafabadi A.H., Aliakbarian B., Arab-Tehrany E., Akbari M., Annabi N., Juncker D., Khademhosseini A. (2015). Hydrogel Templates for Rapid Manufacturing of Bioactive Fibers and 3D Constructs. Adv. Healthc. Mater..

[152] Sun W., Starly B., Daly A.C., Burdick J.A., Groll J., Skeldon G., Shu W., Sakai Y., Shinohara M., Nishikawa M. (2020). The Bioprinting Roadmap. Biofabrication.

[153] Dobashi T., Yamamoto T. (2018). Analysis of Heterogeneous Gelation Dynamics and Their Application to Blood Coagulation. Gels.

[154] Morgan F.L.C., Moroni L., Baker M.B. (2020). Dynamic Bioinks to Advance Bioprinting. Adv. Healthc. Mater..

[155] Khaddour K., Johanns T.M., Ansstas G. (2020). The Landscape of Novel Therapeutics and Challenges in Glioblastoma Multiforme: Contemporary State and Future Directions. Pharmaceuticals.

[156] Hatoum A., Mohammed R., Zakieh O. (2019). The Unique Invasiveness of Glioblastoma and Possible Drug Targets on Extracellular Matrix. Cancer Manag. Res..

[157] Razinia Z., Castagnino P., Xu T., Vázquez-Salgado A., Puré E., Assoian R.K. (2017). Stiffness-Dependent Motility and Proliferation Uncoupled by Deletion of CD44. Sci. Rep..

[158] Radel C., Rizzo V. (2005). Integrin Mechanotransduction Stimulates Caveolin-1 Phosphorylation and Recruitment of Csk to Mediate Actin Reorganization. Am. J. Physiol.-Heart Circ. Physiol..

